# Ethnic differences in parental experiences during the first six months after PICU discharge in Singapore: a qualitative study

**DOI:** 10.3389/fped.2023.1288507

**Published:** 2024-01-05

**Authors:** Pei-Fen Poh, Matthew C. Carey, Joseph C. Manning, Jan Hau Lee, Jos M. Latour

**Affiliations:** ^1^ Faculty of Health, School of Nursing and Midwifery, University of Plymouth, Plymouth, United Kingdom; ^2^ Children’s Intensive Care Unit, KK Women’s and Children’s Hospital, Singapore, Singapore; ^3^ Nottingham Children’s Hospital, Nottingham University Hospitals NHS Trust, Nottingham, United Kingdom; ^4^ School of Healthcare, College of Life Sciences, University of Leicester, Leicester, United Kingdom; ^5^ Paediatrics Academic Clinical Programme, Duke-NUS Medical School, Singapore, Singapore; ^6^ Faculty of Health Sciences, School of Nursing, Midwifery and Paramedicine, Curtin University, Perth, WA, Australia; ^7^ Department of Nursing, Zhongshan Hospital, Fudan University, Shanghai, China; ^8^ School of Nursing, Fudan University, Shanghai, China

**Keywords:** paediatric intensive care, psychological outcomes, paediatric, Post Intensive Care Syndrome—pediatrics, emotional outcomes, social outcomes, ethnicity, health disparity

## Abstract

**Introduction:**

Literature on parental experiences after childhood critical illness has limited representation from diverse ethnic backgrounds. Parents from global ethnic majority groups have reported worst psychological outcomes and required more social support after childhood critical illness.

**Aim:**

To explore the experiences of Chinese, Malay, and Indian parents in the first six months after Pediatric Intensive Care Unit (PICU) discharge of their child in Singapore.

**Methods:**

Sequential semi-structured qualitative interviews were conducted to collect data from a convenience sample of 28 parents at one month (*n* = 28) and at six months (*n* = 22) after their child's discharge from a multidisciplinary PICU. Framework Analysis was adopted as the qualitative analysis strategy. The PICS-p framework was applied *a priori* in the Framework Analysis.

**Findings:**

Three interdependent domains and seven themes framed the 28 accounts in which ethnically diverse parents reported psychological stressors (PICS-p: emotional health), support received (PICS-p: social health) and practical challenges (transitional health) in the first six months after childhood critical illness. In the emotional health domain, parents were affected by different stressors and had different priorities over their child's survivorship. Only Indian parents reported experiences of stress symptoms, at six months post discharge. Malay parents sought solace from their religion more than Chinese and Indian parents. In the social health domain, parents reported various sources and degree of support received. Familial supports were strong across all groups, while community support was more prominent in Malay as compared to Chinese and Indian parents. A third domain, transitional health, was introduced to capture the difficulties parents faced during the transition from PICU survival to home. Parents from non-Chinese families were more likely to report financial challenges and more involvement of spouses after discharge. Complementary medicine or commercial health products were utilized by Chinese and Malay families.

**Conclusion:**

These findings reveal preferred strategies that parents from a global ethnic employ to address the emotional, social and transitional health impacts of their child's critical illness. Future care delivery may consider tailored care plans, communication strategies, and emotional support in PICUs that address the unique ethnic needs of parents during the critical six-months post their child's illness.

## 1 Introduction

Supportive care has advanced, allowing critically ill children to survive with comorbidities that can impact their long-term health (1). Every year, approximately 230,000 children in the USA and 20,000 children in the UK are admitted to a paediatric intensive care unit (PICU) (1, 2). There is evidence on differences in critical illness health outcome among different ethnicities, with worse outcomes in children from global ethnic majority groups (3, 4). The term “global ethnic majority” refers to a recognition of ethnic groups that constitute a significant majority of the world's population, accounting for approximately 85% (5). This term was introduced in 2003 to challenge the negative connotations associated with the concept of “minority” on Black, Asian and Minority Ethnics, to reject the marginalization and disempowerment experienced by ethnic groups (5). Despite their numerical representation, children from global ethnic majority groups have had higher risk of mortality or required cardiopulmonary resuscitation in the PICU when compared to children of white ethnicity (6–8). Furthermore, during a PICU admission, parents from global ethnic majority groups were more likely, when compared with parents of white ethnicity to report instances of discrimination or not being listened to (9).

A mixed-method systematic review, examining the impact of ethnicity on long term parental health outcomes after PICU, found that parents from global ethnic majority groups had worse psychological health outcomes (10). Parents of black ethnicity reported a higher prevalence of post-traumatic stress symptoms in the first three months after PICU discharge, compared with parents of white ethnicity (11). Following discharge, non-Swiss nationalities received more support from social services when compared to Swiss nationals (12). Representation of non-White parents was limited in the qualitative literature and most studies excluded families with limited English proficiency. Studies that included a heterogenous group did not examine the ethnic differences in parental long-term experiences after PICU discharge. In addition, parents from global ethnic majority groups were more likely to drop out from a long-term study (10).

Post Intensive Care Syndrome—pediatrics (PICS-p) is an internationally accepted framework for understanding the long-term health outcomes of critically ill children and their families. This framework recognises the interdependence of PICU survivors and their families (13). Parental health outcomes are categorised into emotional and social health domains. To our knowledge, no studies have examined the ethnic differences in long-term parental health outcomes within the PICS-p framework. Understanding the ethnic differences in parental experiences may facilitate tailored ethnically appropriate interventions to support recovery after childhood critical illness. The Singapore Health outcome After Critical illness in Kids (SHACK) study is a prospective mixed-methods cohort study to examine PICU survivors and their parents' health outcomes in the first six months after PICU discharge (14). This paper reports on the qualitative component of the study with the aim to explore a group of ethnically diverse parents' long-term experiences after PICU discharge, focusing on similarities and differences between the Chinese, Malay, and Indian ethnic groups in Singapore.

## 2 Methods

### 2.1 Study design

A qualitative study design was used to examine the contextual and unique experiences of ethnically diverse parents in the first six months after PICU discharge. The PICS-p conceptual framework was used to guide the design of this study. For a comprehensive understanding of the study methodology, please refer to the published SHACK study protocol (14). The Consolidated Criteria for Reporting Qualitative Research (COREQ) guideline was used in this report (15).

This study adopted an interpretivist standpoint where participants' lived experiences and perceptions are subjective and socially constructed, emphasizing the importance of understanding their individual meanings and interpretations. Through in-depth interviews and thematic analysis, we aimed to explore and interpret the rich and diverse narratives of parents, acknowledging the context-bound nature of knowledge. Furthermore, we acknowledge that there is no single objective reality, but rather multiple subjective realities that emerge through interaction and meaning-making (16). Through this research, we aim to gained insight into the lived realities of parents, recognizing the fluid and evolving nature of their experiences.

### 2.2 Participant and setting

We included parents of children aged between 1 month and 18 years, who were admitted to a 16-bed multidisciplinary PICU for at least 48 h. The annual PICU admission in Singapore is estimated at 900 patients annually, the study setting was a 800 bed paediatric tertiary hospital in Singapore. In Singapore, PICU follow-up care and support are provided by respective medical specialists after hospital discharge and not a dedicated PICU physician. Religious facilities within hospitals are not designated, and families often utilize waiting lounges for prayers. Upon discharge, only medically complex children who require ongoing mechanical ventilation or enteral feeding are prescribed specific equipment such as ventilators, feeding pumps, and pulse oximetry.

In terms of healthcare costs, Singapore operates on a co-payment system. Medical expenses are subsidized based on household incomes to ensure affordability. Citizens can use their Central Provident Fund (CPF) savings to cover a portion of medical fees. Additionally, some families opt for private hospitalization coverage for more extensive healthcare support. We excluded parents of children on palliative services or had a Do-Not-Resuscitate order. Purposive sampling of participants was used to select parents of different ethnicities, representing the ethnic distribution in Singapore. The interviews aimed to explore the experiences of at least 12 parents of Chinese, Malay or Indian ethnicity, four in each group.

### 2.3 Data collection

Serial face-to-face or virtual semi-structured interviews were conducted by the first author (PF) from February 2021 to February 2023. An interview guide was utilized to explore participants’ perceptions of their experiences and support needs as parents (Table 1). Probing questions were employed to facilitate clarification and elaboration of responses. Follow-up interviews were conducted with the same group of parents at one and six months after their child's discharge from the PICU.

**Table 1:** Interview questions.

Interview questions
It's been one/six month since your child was discharged from the PICU. In what ways would you describe the differences in your life compared to before your child's admission to the PICU?
Could you please share your experiences regarding the support you have received during this time? Are there any specific types of support that you wished you had received but didn't?
How do you feel as the primary caregiver of your child after their discharge from the PICU?
Can you please describe the strategies or activities you engage in to help cope with the demands caregiving?
How has your interaction with friends and family changed since your child was discharged from the PICU?
Have there been any other notable changes in different aspects of your life since your son/daughter left the PICU, either one or six months ago? Is there anything else that we have not discussed that you would like to share with me?

The interview guide was developed based on a review of relevant literature and input from clinical and academic experts in paediatric critical care, family-centred care, and long-term outcomes following critical illness. Feedback on the wording of the interview guide was obtained from three parents to enhance clarity and usability. Baseline and clinical characteristics, including demographics (e.g., age, gender, education, religion, and ethnicity), socioeconomic information, and relevant admission data, were collected. Clinical data was obtained from the electronic medical records.

### 2.4 Data analysis

Interviews were audio-recorded and transcribed verbatim by the first author (PF), who incorporated field notes in brackets. The interview data were analysed using a predefined framework based on the domains of the PICS-p framework. The authors PF and MCC read the transcripts and conducted line-by-line coding to identify key themes. The coding of the first five interviews was reviewed by the authors JML, JM, and JH for verification. The interview data were managed using NVivo 12 software (released in March 2020) (17).

The adapted five-stage framework analysis method was used to examine the experiences and support needs of a group of ethnically diverse parents in the first six months after PICU discharge (18). This involved familiarisation with the data, developing a theoretical framework, indexing of the data set, summarizing data in an analytical framework, and synthesizing data through mapping and interpretation. Comparisons between the Chinese, Malay and Indian groups were performed during the final stage of data analysis (Supplementary ESM S1–S3).

### 2.5 Rigour and trustworthiness

The data analysis process involved collaboration among the research team to ensure credibility, originality, resonance, and usefulness (19). Rigour was maintained through immersion in the data and capturing detailed field notes to capture participants' emotions and reactions (PF and MC) (18). Feedback from the research team and participant data validation were conducted to strengthen credibility and resonance (17). The researcher, of Chinese ethnicity, is a PICU nurse pursuing her PhD and holds a Singapore citizenship. She conducted the interviews and utilized field notes to reflect on each interview. To ensure cultural appropriateness during interviews, specific steps were taken. For instance, before conducting a virtual interview with a Malay mother at her home, we verbally confirmed privacy and ensured no men were present, respecting her choice to be without her hijab head covering. To ensure trustworthiness, member checks with participants and triangulation of interview transcripts and field notes were conducted (PF, MC, JM and JML) (20). An audit trail was maintained for accountability and transferability of the findings (21).

### 2.6 Ethical considerations

The study received ethical approval from the SingHealth Centralised Institutional Review Board (CIRB Ref: 2020/2997) and the Faculty Research Ethnics and Integrity Committee of the University of Plymouth (Ref: 2020-2506-1464). Written informed consent was obtained from all participants, and their identities were protected through the use of study numbers and pseudonyms for reporting. Confidentiality was maintained by password-protecting all data files.

## 3 Findings

### 3.1 Characteristics of participants

Twenty-three families of critically ill children with various medical conditions and trajectories completed an interview at one month after PICU discharge. Eighteen families participated in the sequential interview at six months post PICU discharge (Tables 2, 3). Among the 23 ethnic diverse families, there were nine Chinese, eight Malays, five Indians and one Jewish. All interviews were conducted over video calls, except for two, which were conducted face-to-face within the hospital premises. The mean duration of the interviews was 23 min (ranging 9 to 76 min) at one month and 28 min (ranging 7 to 43 min) at six months after PICU discharge. The five parents who did not participate in the second interview at six months was a Chinese father, an Indian mother and three Malay mothers. A minimum of five attempts were made to schedule for an interview at six months and to understand the reason for non-participations. Only one Malay mother cited that she was unable to participate due to her new work schedule.

**Table 2:** Child participant characteristics (*n* = 23).

No.	Child	Sex	Age (years)	LOS Hospital (days)	LOS PICU (days)	Ethnicity	Diagnosis
1	Samuel	M	18	11	2	Chinese	Splenic rupture with background of haemophilia
2	Xinle	F	3	25	4	Chinese	Myocarditis requiring extra-corporeal membrane oxygenation (ECMO)
3	Ethan	M	2	22	4	Chinese	Newly diagnosed neuroblastoma
4	Benji	M	2	26	2	Chinese	Fall from height, critically injured
5	Enning	F	4	7	3	Chinese	Ventricular septal defect post-surgical correction
6	Noah	M	2 months	3	2	Chinese	Congenital heart block
7	Linda	F	9	24	9	Chinese	Motor vehicle accident, critically injured
8	Chris	M	12	5	3	Chinese	Diabetic ketoacidosis
9	Sage	M	14	20	15	Chinese	Pneumocystis pneumonia w background with of leukaemia
10	Niya	F	12	23	23	Indian	Renal failure
11	Jacinta	F	12	7	4	Indian	Newly diagnosed thyroid tumour post-surgical resection
12	Raul	M	8	5	2	Indian	Multisystem Inflammatory Syndrome in Children
13	Mithya	F	7	29	29	Indian	Ventricular septal defect post-surgical correction
14	Ananda	M	8	2	2	Indian	Multisystem Inflammatory Syndrome in Children (COVID)
15	Aish	M	14	18	3	Malay	Newly diagnosed leukaemia
16	Raman	M	1 months	7	3	Malay	Cow's milk protein allergy
17	Nassim	M	4 months	11	3	Malay	Complex congenital heart defect
18	Halim	M	9	23	2	Malay	Respiratory failure with background of cerebral palsy
19	Nurul	F	10 months	17	2	Malay	Atrial septal defect
20	Abdul	M	4	16	8	Malay	Multisystem Inflammatory Syndrome in Children (COVID)
21	Aziqah	F	11	5	2	Malay	Multisystem Inflammatory Syndrome in Children (COVID)
22	Taufiq	M	13	11	3	Malay	Myocarditis
23	Gideon	M	11	17	2	Others, Israeli	Newly diagnosed brain tumour

ECMO, extracorporeal membrane oxygenation; LOS, length-of-stay; PICU, Pediatric Intensive Care Unit.

**Table 3:** Parent participant characteristics (*n* = 23).

No.	Child	Sex	Ethnicity	Parent	Age (years)	Religion	Highest education qualification	Monthly income (SGD)	Time points
1	Samuel	M	Chinese	Father	56	Christianity	Post-graduate	6,000–8,999	1
2	Xinle	F	Chinese	Mother	40	No religion	Polytechnic	3,000–5,999	2
3	Ethan	M	Chinese	Mother	35	Buddhism	Secondary	3,000–5,999	2
4	Benji	M	Chinese	Mother	43	Buddhism	Secondary	3,000–5,999	2
5	Enning	F	Chinese	Mother	35	Buddhism	Undergraduate	1,500–2,999	2
6	Noah	M	Chinese	Father	38	No religion	Post-graduate	6,000–8,999	2
7	Linda	F	Chinese	**Father** and Mother	42	No religion	Undergraduate	6,000–8,999	2
8	Chris	M	Chinese	Mother	39	Buddhism	Polytechnic	<1,499	2
9	Sage	M	Chinese	Father	50	Buddhism	Post-graduate	>15,000	2
10	Niya	F	Indian	**Father** and Mother	75	Islam	Secondary	1,500–2,999	2
11	Jacinta	F	Indian	Mother	39	Islam	Polytechnic	6,000–8,999	2
12	Raul	M	Indian	Father	40	Hinduism	Post-graduate	12,000–14,999	2
13	Mithya	F	Indian	Mother	40	Hinduism	Undergraduate	Not available	1
14	Ananda	M	Indian	Father	44	Hinduism	Post-graduate	3,000–5,999	2
15	Aish	M	Malay	Mother	42	Islam	Junior College	6,000–8,999	2
16	Raman	M	Malay	**Mother** and Father	35	Islam	Polytechnic	3,000–5,999	2
17	Nassim	M	Malay	Mother	43	Islam	Primary	3,000–5,999	2
18	Halim	M	Malay	Mother	40	Islam	Polytechnic	6,000–8,999	2
19	Nurul	F	Malay	**Mother** and Father	31	Islam	Primary	<1,499	1
20	Abdul	M	Malay	Mother	35	Islam	Post-graduate	1,500–2,999	1
21	Aziqah	F	Malay	Mother	35	Islam	Secondary	<1,499	1
22	Taufiq	M	Malay	Mother	50	Islam	Undergraduate	6,000–8,999	2
23	Gideon	M	Others, Israeli	**Mother** and Father	42	Jewish	Post-graduate	6,000–8,999	2

Parent characteristics were reported for parents (bold) who participated in the quantitative survey of the SHACK study.

### 3.2 Overall findings

**Figure 1:**
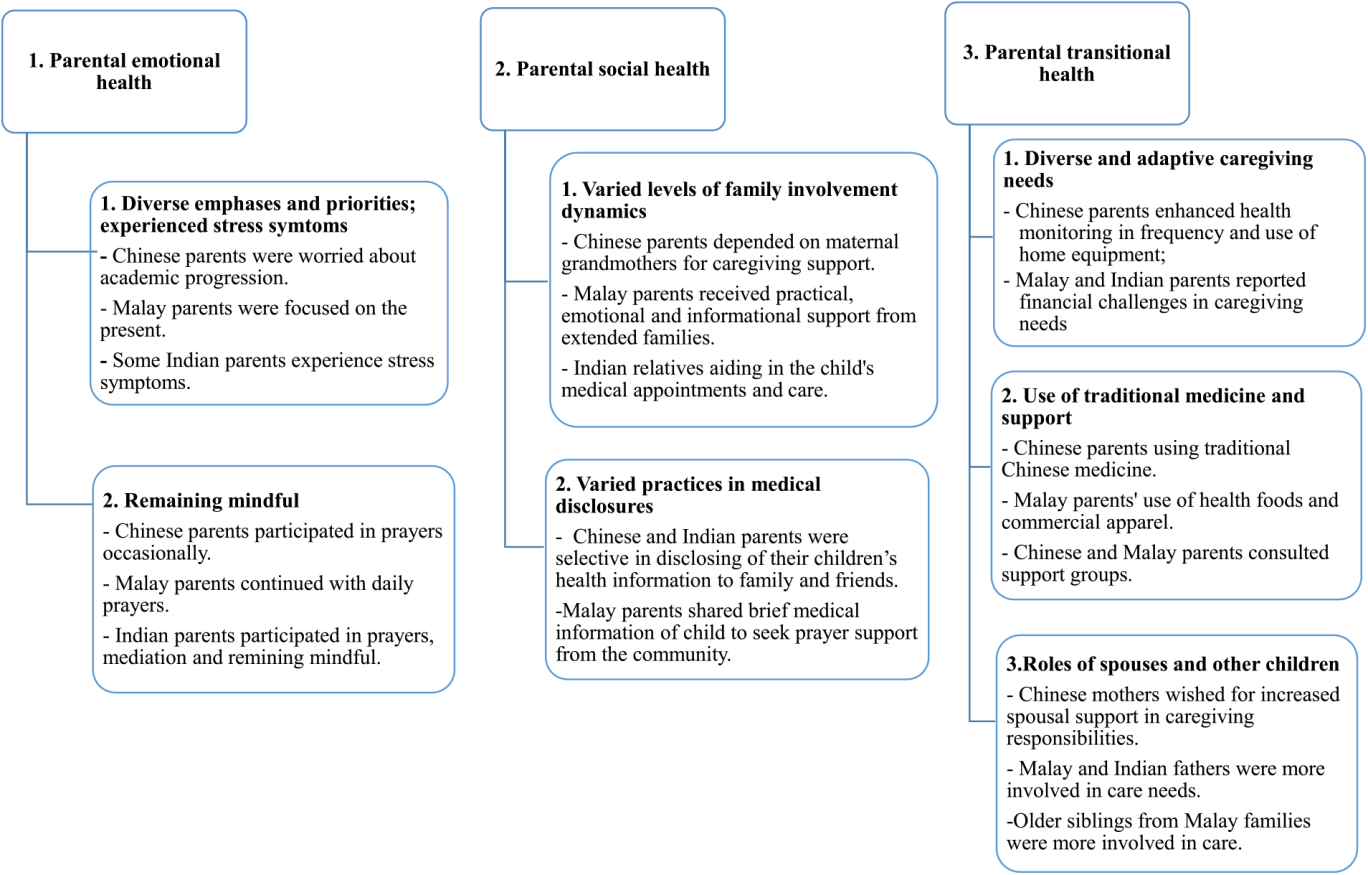
Ethnic differences in regaining normalcy.

Irrespective of ethnic group, for most parents the initial post discharge following their child's critical illness was difficult, with experiences spanning across the emotional and social domains of the PICS-p framework. A third domain, parental transitional health was termed, to capture the challenges parents experienced in transition from PICU survival to home. Regaining of normalcy was prioritised and achieved by most parents, except for those with children who were on active ongoing treatment/conditions following PICU discharge. Figure 1. presents a comprehensive flow diagram that outlines the three domains, seven themes, and nineteen subthemes of these experiences. Notably, the figure also highlights the emergence of ethnic differences, which were further categorized as subthemes to describe the unique experiences of the Chinese, Malay, and Indian groups. These themes are described with pseudo-anonymised participants quotations.

**Figure 2:**
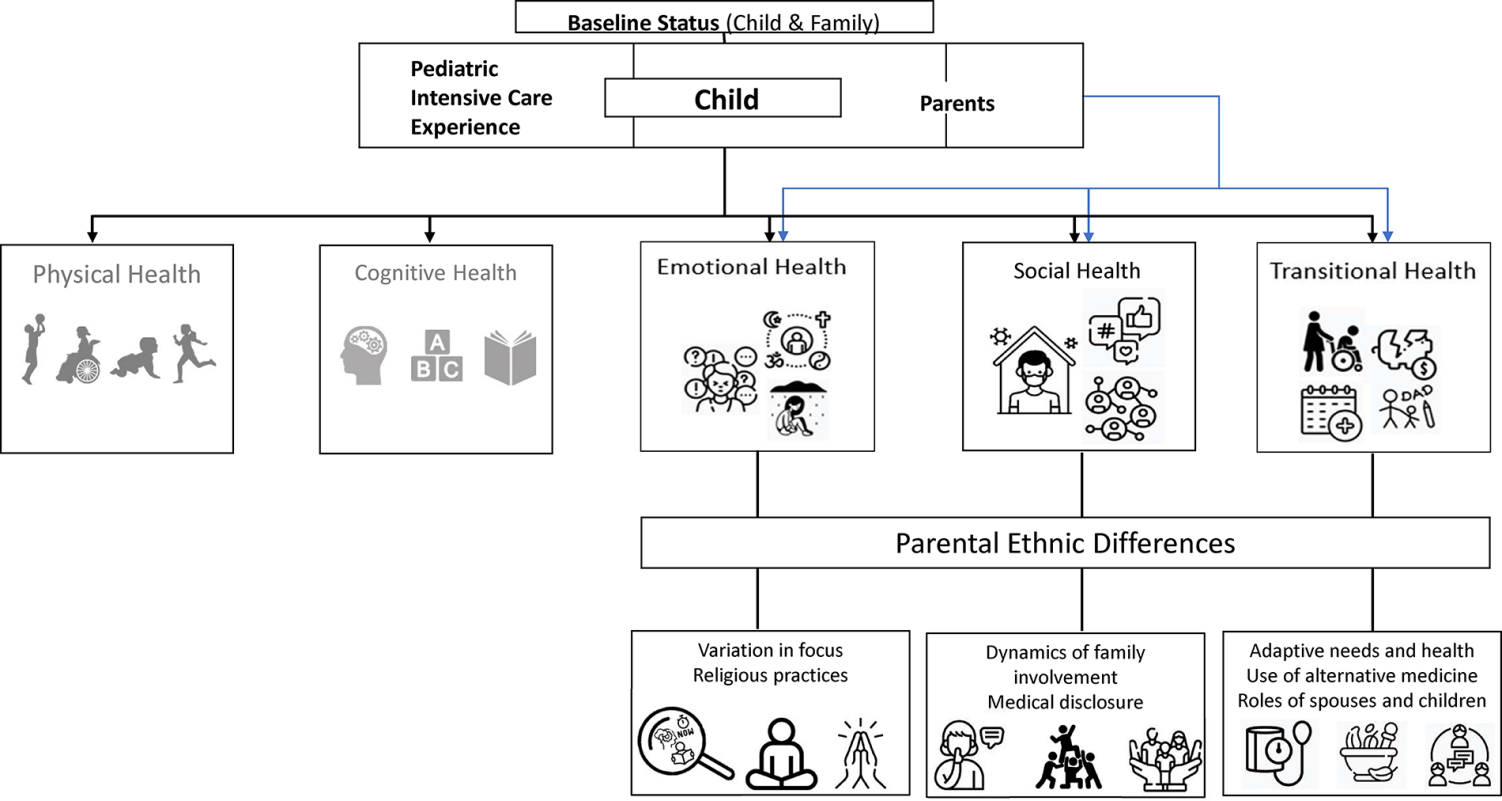
Ethnic differences in parental emotional, social and transitional health. Adapted from the Post Intensive Care Syndrome—pediatrics framework (13).

Figure 2 serves as a visual representation of these distinctive differences within the specific domains of emotional, social, and transitional health. Specifically, we observed variations in how parents from different ethnic backgrounds prioritized their emotional health and utilized religious practices for coping. Socially, there were disparities in the extent of support received from the extended family members based on ethnicity. Moreover, in terms of transitional health, parents from diverse ethnic groups adopted different methods to achieve and maintain their health while adhering to medical follow-ups.

### 3.3 Domain: parental emotional health

#### 3.3.1 Theme: diverse emphases and priorities were observed

Parents had different focuses following PICU discharge. Chinese parents were more future orientated, worrying about PICU readmission and their child's academic progression. Malay families opted to focus on the present, being thankful about their child's survival. Only Indian parents reported stress symptoms at the recall of their child's PICU admission. Chinese parents portrayed a future oriented focus with worries relating to the unknown trajectory of recovery and fear of deterioration of leading to PICU readmission. Although parents felt lucky, that their child survived critical illness, child was recovering well and they had insurance coverage for hospital bills, they reported stressors relating to caregiving needs post discharge. Parents spoke more strongly about worries over academic progression and efforts to help their child catch-up in schoolwork. This was highlighted by the father of Linda (Chinese), aged 42 who stated,

“After *the RTA (road traffic accident), she is not keen to learn because it is very obvious that her Chinese and math have deteriorated a lot. Because if she is unable to catch up with the schoolwork, she might experience more stress in the following years*.” (Father of Linda, Chinese, 6 months,).

Malay parents were focused on the present, acknowledged that they were coping with their child's needs and felt happy with the child's survival and recovery. One respondent, mother of Nassim, (Malay) aged 43, expressed her thoughts, noting,

“*I am doing my very best to stay afloat for the sake of Nassim I cannot remain in that zone, cry and sad. Over little thing I snap.*” (Mother of Nassim, 1 month).

Parents reported taking photos of their child during PICU admission. Strong emotions were evoked when photos were reviewed by accident. However, Malay parents would encourage other parents to take photos for memories about the PICU admission. Parents spoke less about schoolwork and instituted no additional intervention or controls to aid their child's academic progress. Parents spoke more frequently about their trust in healthcare and gratitude towards the healthcare professionals as compared to Chinese parents.

Indian parents more frequently mentioned memories during PICU admission. Parents reported having stress symptoms up to 6 months after PICU discharge, such as “hearing child scream”, tremors and insomnia (mother of Jacinta, aged 39, Indian, 6 months). Stressful symptoms were evoked even after PICU discharge, during medical follow-up in the hospital clinic. Parents described vividly the appearance of their children and how it affected them as they recalled. In an impactful statement, mother of Jacinta, aged 39, expressed,

“*I have added imagination like as if she is not going to wake up then I get teary, I start shaking, it happens for a while and then it subsides on its own. Ya I do start like breathing fast like hyperventilating, it*’*s not for very long.*” (Mother of Jacinta, 6 months).

Mother of Mithya, aged 40, shared how her spouse was traumatised by the screaming of their daughter during a chest tube removal in the PICU, 1 month after discharge.

“When they remove the 2 chest tubes from the surgery, she was screaming in pain. So (on) my husband’s part, he was traumatised with that, until today (1 month) he could still hear her scream.” (Mother of Mithya, Indian, 1 month)

#### 3.3.2 Theme: variations in religious coping strategies, dependency, and beliefs among individuals

Religious practices varied among Chinese, Malay and Indian parents. All Malay parents identified as Muslim and actively engaged in daily prayers even before PICU discharge. Some Malay parents reported an increase in prayer frequency after discharge. Conversely, some Chinese parents stated that they visited the temple more frequently during PICU admission and less after discharge. Additionally, meditation and prayers were observed as coping mechanisms used by certain Indian families during the post-discharge period.

Chinese parents tended to keep to themselves and only disclose a child's health progression to selected families or friend. Parents participated in various activities, such as voluntary work or hobbies, as a form of distraction from the child's health condition. Beliefs and frequency in prayers were different among Chinese parents. Parents tended to pray in the temple to seek divine powers for healing and protection. Mother of Enning (Chinese), aged 35, shared that she uses prayer as a form of coping, “Praying maybe abit, going to the temple.” Some parents prayed as usual, citing conditional prayers as non-sincere. During the interview, mother of Chris, aged 39, offered a thoughtful reflection, stating,

“*We believe that everybody has their destiny in life. If you have to go through certain things in life, you just have to go through it. There will be no difference if you pray a not.”* (Mother of Chris, Chinese, 1 month)

One Chinese couple of different religious beliefs had conflicted ideas about praying in the temple and seeking help from a medium. As shared by father of Noah, aged 38,

“In fact, my mum has asked us to go to those Taoism, those “tangki” (medium), go and ask see whether got offend any spirit. My wife is a catholic, she refused to resort to seek help from the evil force.” (Father of Noah, Chinese, 6 months).

Prayer emerged strongly for Malay parents, parents described prayers as a force to hold the family together, to move the family forward, and a pillar to lean on. Parents had difficulty explaining the calmness they felt after prayers. Some chose “talking to God” over speaking to friends and family (Mother of Nurul, aged 31, Malay, 1 month). An insightful comment came from mother of Aish, aged 42, who shared,

“*That is important, praying is important ah like ah.. it keeps the family bonded and then I think arh it*’*s the internal strength, it helps in the internal strength for both patient and the caregiver, arh we rely and fall back onto our religion.*” (Mother of Aish, Malay, 1 month).

Prayers of the community were cited as important, where the Muslim community collectively prays for the ill during mosque sessions. Indian parents participated in various forms of coping behaviours and emphasized on their personal preference. Some parents focused on facts, remained optimistic, being respectful of the situation, letting time pass and not looking back. During the interview, father of Raul, aged 40, shared,

“*We do our best, that*’*s what we do all the time, be respectful of the situation, not to let it affect you too much. Look at the positive side that he is better*.” (Father of Raul, Indian, 1 month).

Some parents participated in prayers together with their children and some used meditation to help them refocus on the situation.

### 3.4 Domain: parental social health

#### 3.4.1 Theme: varied levels of family involvement dynamics

Parents reported different degrees of support received. Chinese parents most commonly receive support from maternal grandmothers. Malay and Indian families received support from other family members, such as their siblings, in addition to grandmothers. Chinese grandmothers support families by taking charge of the mother's household responsibilities, such as cooking and supervision of other children. Over time, some grandmothers of children requiring long-term medical needs, moved in with the family in continuation of support. Mother of Xinle, aged 40, shared on the help she has received, stating,

“*after the discharge, she stayed another two more weeks. She (grandmother) actually prepared some food, she was mostly in-charge of the daily meal…when my mum was here I totally let my mother fully control. Then when I go back to office, once or twice a week I need to go back. My mother will help me monitor (her health)*.” (Mother of Xinle, Chinese, 1 month).

Malay grandmothers were involved as companions and would regularly visit the family to provide emotional support to the parents, child survivor and siblings. Mother of Raman, aged 35, highlighted the role of grandparents, stating,

“*No.. nobody come and help me, they just come to visit the grandchildren… to help me out, no lah. my parents are old already. No need lah*.” (Mother of Raman, Malay, 6 months).

Malay parents' siblings made efforts to remain in touch and provided support by sharing health information and cooking recipes to enhance the child's appetite following chemotherapy. Indian parents identified practical assistance from their siblings as a distinct form of support. For instance, some Indian parents reported receiving help from siblings in transporting their child between appointments as a means of sharing the financial burden and addressing limitations on work leave. Mother of Jacinta, aged 39, emphasized the significance of family support, revealing,

“*We are actually taking turns. My brother is helping me out as well. Monday Tuesday and Wednesday my brother brought her then tomorrow I will be bringing her, otherwise I am already going on no pay leave because of all these appointments I have used up all my leave, so everybody is chipping in to help a bit lah*.” (Mother of Jacinta, Indian 1 month).

Despite this type of support being perceived as valuable, some parents expressed a reluctance to impose on others and preferred to maintain self-reliance in coping with the situation. Gaining insight into the distinct social support systems available to each ethnic group can enhance the identification and utilization of potential support networks in assisting parents during and after PICU discharge.

#### 3.4.2 Theme: varied practices in medical disclosures

Parents reported different levels of medical disclosure towards external families and friends. Chinese and Indian parents tended to only disclose about their child's medical condition to close family members. Malay families appreciated community support and prayers. Chinese parents tended to restrict the disclosure of their child's experiences to a selective group of individuals due to their apprehension towards receiving negative comments. The parents perceived that their friends might not fully comprehend the complexity of the PICU experiences and were reluctant towards repetitive explanations. One respondent, father of Sage, aged 50, expressed his reluctance to engage with friends, noting,

“*We do not want to trouble friends, family or neighbour to worry, things that has happened we can*’*t really tell people because they can*’*t get anything, they might tell you that Sage will get well, I think it*’*s just consoling, and we do not know if that will really happen*.” (Father of Sage, Chinese, 1 month).

Some parents revealed their preference for information over generic expressions of support. Parents were careful in disclosing child's illness severity and comorbidities to their elder parents, in the fear of grieving them.

Malay parents were willing to provide brief information about their child's health to gather support for community prayers from individuals and in the mosque. However, parents remained vigilant on possible negative comments over the child's condition and recovery. During the interview, mother of Abdul aged, 35 expressed her religious beliefs and gratitude, sharing,

“*You know we believe prayers from the people make more difference than one person praying. A lot of people are praying for him. Some of our friends go to the sacred place to pray for Abdul as well. I know for our religion, He is there, He never leave us. That*’*s why prayer really helps*.” (Mother of Abdul, Malay, 1 month).

Indian parents preferred to remain self-sufficient and were more mindful about burdening family and friends. Medical information was given to external family members who were involved in the care of the child. Mother of Jacinta, aged 39, expressed her thoughts, noting “*Some close friends know, I didn't share with a lot, just some close friends and my (work) superior*.” (Mother of Jacinta, Indian, 1 month). The recognition of parents' diverse preferences for privacy following PICU discharge highlights the critical need to acknowledge and respect cultural differences. This understanding empowers healthcare professionals to offer culturally sensitive support, thereby enhancing the overall well-being of the family.

### 3.5 Domain: parental transitional health

#### 3.5.1 Theme: diverse caregiving needs and adaptive strategies to promote health and well-being

Parents initiated different levels of health monitoring after PICU discharge. Chinese parents monitored their children's health at home more frequently than other parents. These included heart rate, blood pressure and blood sugar monitoring. Additional monitoring was instituted for early detection of health deterioration. Malay parents did not share about additional health monitoring at home. One shared that they would seek help from their family doctor rather than self-medicating in the fear of PICU readmission. Overall, there were more reports of financial difficulties among non-Chinese families.

More Chinese parents were worried about deterioration and had taken on additional vital signs monitoring, such as blood pressure, heart rate and pulse oximetry at home. Mother of Xinle, aged 40, shared, “*Then I went to buy the equipment to test her heartbeat and blood pressure. Every day at least need to test twice lah*.” (Mother of Xinle, Chinese, 1 month). In terms of employment, most mothers had taken time-off from work; all but one has returned to work at six months after PICU discharge.

Malay parents did not mention additional vital sign monitoring but had a lower threshold to seek help when the PICU survivor was unwell. Parents also reported difficulty in managing the daily household chores due to the new care needs of the PICU survivor. One family was receiving financial help in the caretaking of other young siblings to facilitate the homecoming of the sick child. Mother of Raman, aged 35, expressed her financial difficulties arising from the cost of the unfunded prescription milk formula for her son, stating,

“*The milk is not funded all on our own. When I go to the hospital, just get one tin to put in my bills. Other than that, we buy everything on our own. That*’*s about $400 a month*.” (Mother of Raman, Malay, 1 month).

Following PICU discharge, most children did not require any further support, apart from taking prescribed oral medications, attending follow-up appointments, and complying with the prescribed restrictions on physical activity. One Indian family reported that they had returned to their usual routine, except for the need for peritoneal dialysis. However, two families faced more severe financial challenges due to the medical demands arising after the PICU discharge. The emerging theme of financial challenges was highlighted by mother of Jacinta, aged 39, who expressed,

“*It*’*s just the finance side and the work side it*’*s like this I really don*’*t know how, I cannot afford to keep losing money at work and also at the insurance side I am losing money and here I am incurring medical bills. Ya (nervous laugh), so that*’*s the only stress I am really facing at the moment*.” (Mother of Jacinta, Indian, 6 months).

The recognition of parents' varying levels of financial challenges following PICU discharge underscores the significance of targeted assistance to alleviate financial burden and improve health monitoring strategies for families from diverse backgrounds to enhance post-PICU outcomes.

#### 3.5.2 Theme: Use of traditional medicine, commercial health products and support

Expectations from support groups were different. Two Chinese parents shared that support groups were not helpful in understanding their situation and wished for information that was directed at their situation (roles of parents in caregiving, unique experiences of parents whose children had attempted suicide). Two Malay mothers shared about the overseas support group they had joined and were satisfied with the content they received, despite being a non-local support group. One Malay and one Chinese parent shared about their use of alternative medicine or product on their children to complement existing therapy. In contrast, Indian parents did not mention the use of alternative medicine, products, or involvement in a support group.

Chinese parents shared that the support group they attended was not helpful as they preferred to talk about strategies to handle day-to-day caregiving issues, than to explore the feelings about their caregiving experiences. One participant, mother of Benji, aged 43, reflected on her experience at a support group, saying,

“*it*’*s very important for a parent support group like parents can come together. They will know they are not alone. For me at this point in time, I felt that there are some things you want to tell people, but you feel that people won*’*t understand because they are not at that stage*.” (Mother of Benji, Chinese, 6 months).

One family sought help from traditional Chinese medicine which performed regular accu-massage and acupuncture on child. The parents believed that child's condition improved following visits to traditional Chinese medicine (TCM) practitioner. Father of Noah, aged 38, recalled, “*The effect of the TCM is quite obvious. The heart rate will remain low for the next 3 to 4 days after the acupuncture*.” (Father of Noah, Chinese, 6 months).

Malay parents promoted recovery by seeking recipes of health foods and the use of adjunct products such as commercial compression trousers to improve lower limb circulation. Mother of Taufiq, aged 50, recalled,

“*When he was in the hospital, I research about his infection and what are the food, he can eh… in his system to shoo away all the infection fast. Medicine wise I can*’*t, leave it to the doctors, antioxidant I will give it to him*.” (Mother of Taufiq, Malay, 1 month).

Parents reported joining overseas support groups to understand more about the child's condition and the experiences of other parents. Mother of Nassim, aged 43, shared her challenges on a local support group, saying,

“*I joined this Down Syndrome support group, which is not from Singapore. I think Singapore is a bit conservative. There*’*s nothing I can find from Instagram or Facebook. I joined this from America, they give a lot of advice and support, you know*” (Mother of Nassim, Malay, 1 month).

The internet offers access to established support groups, but recognizing diverse needs, local support groups may better cater to parents. Open communication with health professionals is crucial regarding alternative medicine and health food to ensure safety and balanced diets.

#### 3.5.3 Theme: role of spouses and children in meeting the care needs of PICU survivors

There was more mention of fathers' involvement in non—Chinese families. Overall, only Malay mothers mentioned about keeping the peace of their relationship with the spouse by being appreciative and helping each other. Unlike Chinese and Indian, there were no complaints of Malay fathers over issues such as level and involvement in childcare or with incomplete information after medical appointments. Active sibling involvement was more evident in Malay families, where older siblings provided companionship or supervision while mothers took a break or was working on another task.

Some Chinese fathers became more expressive and engaged with their children following their hospitalization in the PICU, in contrast to their pre-admission involvement. In some Chinese families, there were different opinions between parents in terms of their child health's needs. Mother of Chris, aged 39, recalled, “*The father will say just one two bite is fine, so we always have this small argument over these one two bites (laugh)*.” (Mother of Chris, Chinese, 1 month). In another Chinese family, Mother of Ethan, aged 35, had wished for more support from her spouse,

“Hope spouse could attend doctor review and always get update on child’s latest status, and more accompany the child to understand that what our child facing now for example, communication, feeding and behaviour.” (Mother of Ethan, Chinese, 6 months)

In addition, Chinese siblings were largely empathetic and accommodating of their PICU-surviving sibling, often tolerating their occasional negative behaviours.

There was greater emphasis on the collective experience of Malay families in navigating the post-PICU journey. Fathers were reported to be more empathetic and assumed specific caregiving responsibilities after discharge, such as helping the child with the activities of daily livings. Mother of Halim, aged 40, vividly recalled,

“my husband also participate in all the caregiving. He is a hands on father lah, he help to do the suctioning and all that also. he wants to learn everything, all this continuous feeding.” (Mother of Halim, Malay, 1 month)

Siblings in Malay families were more involved in the day-to-day care of the PICU survivor, including providing companionship and occasionally babysitting. Most mothers acknowledged the importance of maintaining a harmonious and peaceful home environment, and they expressed gratitude for the support and presence of their spouses and children. Parents shared on the strategies they used to function as a team and to mitigate conflict during stressful situations.

Indian parents shared about the different caregiving roles, with mothers being the primary caregiver while fathers contributed by accompanying their child for medical appointments and providing companionship to the child. Mother of Jacinta, aged 39, reflected on the changes, saying,

“*At home wise, he would sit down, play with her and talk to her and it*’*s more, now he is more understanding towards her compared to last time. Last time, he was a very strict father. Now he has mellowed down.*” (Mother of Jacinta, Indian, 1 month)

Similarly, Father of Niya, aged 75, previously responsible for transporting Niya to and from haemodialysis sessions, saw a transition in caregiving duties to Niya's mother after her shift to peritoneal dialysis.

*“Now because of the PD, we have to make sure that the tube is properly disinfected and there is not infection of the tube. In that sense, more work for my wife lah.” (Father of Niya, Indian, 6 month)*

The varying level of family engagement and potential role adjustments in primary caregivers were evident in the first six months after PICU discharge.

## 4 Discussion

In this study, we included 23 families from the global ethnic majority group in an attempt to bridge the gap between the scarce literature in examining the experiences of a diverse ethnic group in the study population. Purposive sampling ensured a sample of parents representing the ethnic distribution of Chinese, Malay, and Indian in Singapore. By adopting a qualitative design and employing sequential serial interviews, we obtained a comprehensive understanding of parental experiences over the first six months following their child's discharge from the PICU. Parents from the Malay ethnic group reported the highest attrition, completing one-out-of-two interviews in the first six months of discharge. We examined the differences in parental experiences and support needs across the emotional, social, and transitional health domains of the PICS-p framework. Overall, parents of different ethnic groups use different coping strategies, received various degrees of social support, and exhibited unique help-seeking behaviours. The diverse cultural practices, values, and norms, as well as the community dynamics associated with various ethnicities, may have contributed to the variations in parental experiences during the first six months following their child's discharge from the PICU.

Coping behaviours following a child's critical illness vary among parents from different ethnic groups, reflecting their unique cultural context. Malay parents who were deeply rooted in the Islam beliefs relied on their religion as a source of solace, finding strength through individual, familial and community prayer (22). Indian parents drew upon spiritual beliefs such as Hinduism, utilized prayers and meditation for self-reflection, inner peace, and emotional healing. Whilst Chinese parents did not prominently emphasize religious practices, some occasionally partook in certain religious rituals or visited temples during stressful times. These differences highlighted the different ways ethnic background influences parental coping behaviours after PICU discharge. In another study, parents of Islamic faith reportedly perceived higher social support caring for technologically dependent children (23). Malay parents’ religious devotion provides a framework for understanding life's fragility, fostering trust, and psychological dependence. Indian parents derive strength from their spiritual heritage, embracing mindfulness to cope with challenges. Chinese parents, while not religiously orientated, were focused on the future, coping by anticipating possible challenges. Other investigators have observed that Islamic beliefs and practices may have a communal effect, with parents reporting higher perceived support in medically complex children (23). Recognising these unique coping strategies emphasizes the importance and the need for healthcare providers to provide culturally sensitive care in supporting families after PICU discharge, noting that negative religious coping was associated with a higher level of psychological distress in parents of children with cancer (24). By understanding the preference for the use of religious and spiritual practices, healthcare providers can effectively assist parents on their journey to their child's recovery (25).

Parents of various ethnic backgrounds received different social support following their child's critical illness. Social support has been demonstrated to play a crucial role in helping and promoting well-being for individual caregivers, the patient, and the whole family unit (26). One study examining resilience and psychosocial morbidity of parents after PICU discharge, reported that parents of different ethnic backgrounds received differing degrees of family support. Latino families reportedly received more support from their external family as compared to White parents. Having more support may have consequently resulted in a lower lever of stress reported by Latino parents (27). In Australia, support from grandmothers were also reported where parents recalled grandparents travelling from across the countries to provide support post PICU discharge (28, 29). In Singapore, higher perceived support was predictive of lower parental caregiving stress in technologically dependent children requiring mechanical ventilation or nasogastric tube feeding (23). Therefore, enhancing parental perceived support is important to their well-being. These diverse forms of social support reflected the cultural nuances within each ethnic group. Understanding the specific types of social support needs the Chinese, Malay and Indian may facilitate matching unique requirements of each group.

Ethnic differences in help-seeking behaviours among parents highlight diverse approaches to support and involvement within different cultural contexts. Unlike a previous study on Asian parents in which parents adopted passive coping strategies such as avoidance and withdrawal rather than problem focused coping, this study sheds light on the varied responses and cultural nuances within the studied population (23). Chinese and Malay parents in this study mentioned the use of complementary methods to improve recovery after PICU discharge. Some Chinese parents sort help from traditional Chinese medicine, using acupuncture to improve the “strength” of organs to improve overall health. Some Malay parents used non-medical pressure trousers to improve blood circulation and explored diets high in antioxidants to promote recovery. The use of complimentary methods remains popular among patients and providers seeking a holistic approach to paediatric care (30). However, there is limited literature on the use of complimentary methods such as acupuncture, dietary supplements, or products on the care of children after critical illness. Future research may investigate the impact of complimentary methods and parental locus of control in helping their children recover after critical illness. The lack of adequate pediatric support groups in Singapore, specifically catering to PICU patients and those with ongoing medical needs post-discharge, represents a significant challenge. Scarcity of these support group contributes to a gap in crucial resources and assistance for families navigating the challenges following childhood critical illness. Our findings support the observation that support groups for PICU survivors are limited in Asia region and suggests that formation of such groups may be beneficial to families (23).

Large-scale migrations have resulted in multi-ethnic communities even within previously homogenous countries (31). As more critical care societies such as the European Society of Intensive Care Medicine and Society of Critical Care Medicine and the Society of Critical Care Medicine, emphasize research equity, inclusiveness, and diversity, understanding multiculturalism in paediatric critical care is crucial (32, 33). Our study aligns with these principles by exploring the experiences of both majority and non-majority populations, providing a holistic representation of the epidemiology of paediatric critical care health. Although conducted in Asia, this study offers insights into multiculturalism in an international context. Singapore's multi-ethnic cosmopolitan nature is reflected in the findings, which highlight a blend of cultural hybridity, combining Western modernity with Asian values (34). By encompassing multicultural perspectives, the findings enhance our knowledge and promote culturally sensitive care for families in multicultural environments after childhood critical illness.

### 4.1 Strengths and limitations of the work

This embedded qualitative component of the SHACK study allows a comprehensive understanding of ethnic differences of long-term experiences after PICU discharge. The sample size exceeded the initial target of 12, with 23 parents interviewed, providing a richer and more diverse dataset. The use of the sequential interview approach was a novel aspect of the study, shedding light on the evolving trajectory of recovery. This study demonstrates a commitment to rigour through sequential serial interviews, expert consultation with team members well-versed in qualitative methos, diligent use of field notes, and rigourous multiple deliberations among team members.

However, there are some limitations that need to be explicated. Despite efforts to reach out, five families did not complete the second interview, potentially impacting the completeness of the data. The findings, specific to a state-sanctioned multi-ethnic society, offer valuable insights into cultural differences in long-term care experiences, but generalizability to other settings may be limited. There was limited exploration of the relationship between participant's socio-economic background on the experiences of parents following childhood critical illness. Moreover, the study focused solely on parent's perspectives, excluding the viewpoints of the child survivor, siblings, healthcare providers and other stakeholders, which could have provided a more comprehensive understanding of long-term experiences after childhood critical illness.

### 4.2 Recommendations for further research

Further research is recommended to enhance our understanding of long-term experiences after PICU discharge. Replicating the study in diverse cultural, involvement global ethnic majority groups would provide a broader perspective and enhance generalizability. Additionally, investigating the reasons behind non-completion of the second interview by families would improve study design and participant engagement. Lastly, exploring the long-term impact of ethnicity on parental health outcomes beyond six months would enable the development of culturally sensitive interventions and support programme.

### 4.3 Implications for policy and practice

The evidence of the global ethnic majority in long-term experiences after childhood critical illness highlights the need for healthcare policies and practices to be sensitive to diverse ethnic groups. It is important to address varying levels of support from extended families, reliance on religious coping, and the lack of local support groups. Policy interventions should focus on promoting culturally appropriate support systems and resources to enhance the quality of care and support provided to families, improving long-term outcomes and well-being. Healthcare practices should emphasize the recognition of different ethnicities and their specific needs starting from the PICU admission. Taking cultural diversity into account can aid tailoring care plans, communication strategies, and emotional support to enhance outcomes for patients and their families.

## 5 Conclusion

This study addresses critical gaps in the literature by providing novel and contemporary insights into parental experiences in the first six months following their child's critical illness from a sample of global ethnic majority. By employing an analytical approach utilising the PICS-p framework and ethnic stratification of the sample, similarities, and differences in relation to how the critical care encounter and survivorship is experience has been identified. These findings identify preferred strategies employed by parents to mitigate the emotional, social, and transitional health impact of their child's critical illness. This study underscores the importance of incorporating ethnicity as a crucial factor in shaping PICU practices and guiding future research endeavours.

## Data Availability

The original contributions presented in the study are included in the article/Supplementary Material, further inquiries can be directed to the corresponding author.

## Supplementary material

Supplementary ESM 1 Framework analysis, Stage 3, example of coding process.

Supplementary ESM 2 Framework analysis, Stage 4.

Supplementary ESM 3 Sample of data analysis.
