# Supplementary material for: Ethnic differences in parental experiences during the first six months after PICU discharge in Singapore: a qualitative study
Source: Front Pediatr. 2024 Jan 5;11:1288507. doi: 10.3389/fped.2023.1288507 (PMC10796750; doi:10.3389/fped.2023.1288507)
Supplement: Supplementary ESM 1 — Framework analysis, Stage 3, example of coding process. [file Table1.pdf]

Emotional Health B.Ethnicity Coping Strategies-Chinese	12	26
Emotional Health B.Ethnicity Coping Strategies-Indian	8	13
Emotional Health B.Ethnicity Coping Strategies-Malay	13	24

016_Xinle_Chinese
Actually got sign up a class with my friend, that kind of class is not like dancing class but its like therapy. Dancing therapy class. Its like part of like relaxation and some of the body flow movement. I find something quite good.

026_Benji_Chinese
(volunteering) you focus the energy somewhere else lor, yea and then when you are busy, your mind is not on him. you are busy with the events, you are busy with the activities being done the mind is not always on him. it distract you for awhile, take you away from the problem for awhile.

001_Niya_Indian
yea I I always uhm believe in uhm, power of spiritual tsk spiritual power lah you know, you know whatever religion you belong to ah, maybe will help you in the hour of this hour of sadness, so... so I always welcome that you see also. Beside of taking modern medicine, we must also believe the power of the unseen, you know whatever religion you belong to that there is that have to come from somewhere

109_Mithya_Indian
I think its a very good pillar of support, for us we did pray a lot, we did put a lot of prayers near her bed, in fact she slept better with the prayers on. Yea, she always ask for prayers then she is able to sleep.

012_Nassim_Malay
he is consistent, full time now I would say. full time.. yea 5 times a day he never miss, 5 times a day. I would say Nassim is a blessing from God above.

006_Aish_Malay
arh yes, that is important, praying is important ah like ah.. it keeps the family bonded and then I think arh it the internal strength, it helps in the internal strength for both patient and the caregiver, arh we do rely and fall back onto our religion.
