# Supplementary figures and images for: Ethnic differences in parental experiences during the first six months after PICU discharge in Singapore: a qualitative study
Source: Front Pediatr. 2024 Jan 5;11:1288507. doi: 10.3389/fped.2023.1288507 (PMC10796750; doi:10.3389/fped.2023.1288507)

ESM 2. Framework analysis, stage 4, example of categories within a working analytical framework


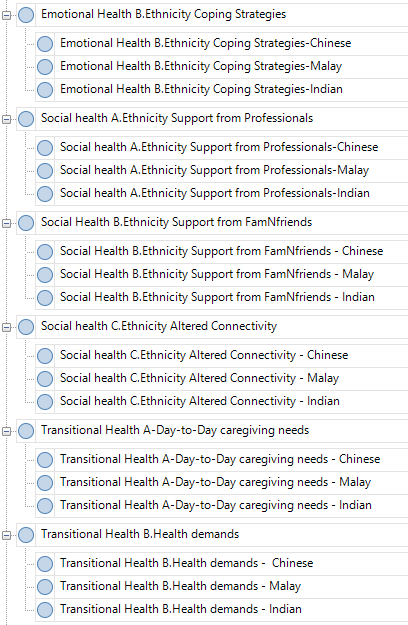

Supplement: Supplementary ESM 2 — Framework analysis, Stage 4. [file Table2.docx]
