# Supplementary material for: Ethnic differences in parental experiences during the first six months after PICU discharge in Singapore: a qualitative study
Source: Front Pediatr. 2024 Jan 5;11:1288507. doi: 10.3389/fped.2023.1288507 (PMC10796750; doi:10.3389/fped.2023.1288507)
Supplement: Supplementary ESM 3 — Sample of data analysis. [file Table3.docx]

ESM 3. Sample of data analysis: Themes, Subthemes and Quotations of Chinese, Malay and Indian parents.

| Theme | Theme descriptor | Subtheme | Descriptor for Chinese parents | Descriptor for Malay parents | Descriptor for Indian parents |
| --- | --- | --- | --- | --- | --- |
| 1.1. Variations in areas of focus and priorities among parents | Parents had different focus following PICU discharge. Chinese parents were more future orientated, worrying about PICU readmission and academic progression.  Malay families opted to focus on the present, being thankful to about child’s survival.  Only Indian parents reported stress symptoms at the recall of their child’s PICU admission. | Chinese parents were worried about academic progression.  Malay parents were focused on the present.  Some Indian parents experience somatic stress symptoms. | Parents portrayed a future oriented focus with worries relating to unknown trajectory of recovery and fear of deterioration of current health leading to PICU readmission. Although parents felt lucky that child survived critical illness, recovering well and had insurance coverage for hospital bills, they reported stresses relating to caregiving needs post discharge. Parents spoke more strongly on worries over academic progression and efforts to help child catch-up in schoolwork “*after the RTA she is not keen to learn because it is very obvious that her Chinese and math has deteriorated a lot. because if she is unable to catch up with the schoolwork, she might experience more stress in the following years*.” (034_Linda at 6 month). There was less mention of reminiscing of child’s PICU admission and gratitude towards healthcare providers. | Parents were focused on the present, acknowledged that they were coping with the child giving needs and felt happy that with child’s survival and recovery. “*I have to learn, I am very scared you know I am not a nurse even injection for myself will be very very painful, I am scared to carry out injection at home but I have to be brave for him, he is brave. I don’t have a choice, i have to learn slowly then he can go home..*” (093_Abdul at 1 month). Parents reported taking photos of child during PICU admission. Strong emotions were evoked when photos were reviewed by accident but would encourage parents to take photos for memories. Parents spoke less about schoolwork and no additional intervention or controls to aid child’s academic progress. Parents spoke about their trust in healthcare and gratitude towards the healthcare professionals. | There were more mentions of memories during PICU admission. Parents reported having stress symptoms up to 6 months after PICU discharge such as “hearing child scream”, tremors and insomnia. Stressful symptoms were evoked even after PICU discharge, during medical follow-up in the hospital clinic. Parents described vividly the appearance of their children and how it affected them as they recalled “*I have added imagination like as if she is not going to wake up then I get teary, I start shaking, it happens for a while and then it subsides on its own. ya i do start like breathing fast like hyperventilating, it’s not for very long.*” (071_Jacinta at 6 month) Parents encouraged child to return to schoolwork in preparation for school returning. Parents were appreciative with the care they received and the health care professionals they have encountered. |
